# The Discovery of Anæsthesia

**Published:** 1877-06

**Authors:** J. Marion Sims

**Affiliations:** NEW YORK.


### ARTICLE III.

### *The Discovery of Anæsthesia.*

BY J. MARION SIMS, M. D., NEW YORK.

Long before the days of Horace Wells and of Morton and Jackson, we were on the eve of the discovery of anæsthesia. In 1790, Priestley discovered nitrous oxide gas. In 1799, Sir Humphrey Davy experimented with it, and in 1800, he published his *Researches, Chemical and Philosophical, chiefly concerning Nitrous Oxide Gas and its Respiration*, in which he says, "As nitrous oxide, in its extensive operations, appears capable of destroying physical pain, *it may probably be used with advantage during surgical operations*, in which no great effusion of blood takes place." Sir Humphrey Davy had inhaled the gas repeatedly for headache and other painful affections, and finding relief for the time, he suggested its use as an anæsthetic in surgery; and if he had been a surgeon, there is no doubt he would have used it as much. But his great idea was lost to the world for more then forty years.

There are four claimants for the honor of the discovery of anæsthesia, viz: Crawford W. Long, of Athens, Georgia; Horace Wells, of Hartford, Ct.; W. T. G. Morton and Charles T. Jackson, of Boston.

I propose to give a plain statement of facts bearing on the question, leaving the reader to draw his own conclusions.

The claims of Long have never been fairly stated in connection with those who came after him. I am ashamed to say I was wholly ignorant of them until a very recent day, and I believe that the great mass of the profession are in the same category with me. I became acquainted with the facts of Long's labors by mere accident.

In October, 1876, Dr. P. A. Wilhite, of Anderson, S. C., came to New York to consult me about the health of his daughter. Her case required a surgical operation, and it was necessary for her to take ether, which was given by Dr.

Harry Sims. After the operation was over, and while we were waiting to see our patient fully restored from the effects of the anæsthetic, the conversation naturally turned upon the wonders of anæsthesia, when Dr. Wilhite said, "Doctor, I assisted at the first operation ever performed under influence of ether." I said, "but how could this be when you have never been in Boston, and the first operation ever performed under ether was by Warren, of Boston, in October, 1846, or as some claim, by Marcy, of Hartford, in January, 1845." Dr. Wilhite then told me that he had assisted Dr. Crawford W. Long, of Georgia, in extirpating a tumor from the neck of Mr. Venable, in March, 1842, while he was completely anæsthetized by the inhalation of sulphuric ether—that Mr. Venable was as profoundly anæsthetized as the patient then lying before us—and he also said that he had assisted Dr. Long to operate on other patients under the influence of ether in 1843 and '44, while he was a student of medicine in Dr. Long's office. He declared that Long was the real and original discoverer of anæsthesia, and he believed he would be so acknowledged if all the facts in the case were fully set forth.

He further said that he presumed that he (Dr. Wilhite) was the first person who had ever profoundly etherized any one—and it was under these circumstances. Dr. Wilhite says that from the time he was ten years old (1832,) he was familiar with the use of ether by inhalation as an excitant; that the boys and girls in his neighborhood near Athens, Georgia, were in the constant habit of using it; that there was hardly ever a gathering of young peaple that did not wind up with an ether frolic. Old-fashioned "quiltings" were very common in his day and time, and in the evening the boys and young men would go to these for the purpose of a dance or an ether frolic.

On one occasion, he met several young people at Mr. Ware's, about five miles west of Athens, at a quilting. The girls and boys all finished the evening by inhaling ether. Some would laugh, some cry, some fight, and some dance, just as when nitrous oxide gas is inhaled. It was in the fall of 1839. Wilhite was a romping boy of seventeen. All the boys and all the girls had inhaled the ether, some some of them more than once. They were looking around for new subjects for it, when Wilhite saw a negro boy at the door, who seemed to be enjoying the sport. Wilhite invited him to come in and try the ether. He refused. Other young men then insisted on his taking it. He refused again in a most positive manner; whereupon some of the thoughtless young men caught hold of the boy, and called Wilhite to give him the ether. He struggled violently, but they threw him down and held him there while Wilhite poured out some ether on a handkerchief, and pressed it firmly over his mouth and nose. He fought furiously. They persisted, thinking it was great fun. After a long struggle, the boy became quiet and unresisting, The young men then let him alone. They were greatly surprised that he did not get up immediately and say or do some foolish thing for them to laugh at. He lay quietly, and with stertorous breathing. They tried to arouse him, but could not. They then became greatly alarmed, and sent one of their number on horseback for Dr. Sydney Reese, at Athens. five miles distant. The messenger rode with all possible speed. He fortunately found Dr. Reese at home, who lost no time in going to Ware's. On his arrival, he found the negro lying on his back still soundly asleep. The young ladies had left the frightful scene. Young Wilhite, and his principal accomplice, thinking that they had in mere play murdered a fellow being, were so much alarmed, that they contemplated making their escape from the country; but the timely arrival of Dr. Reese soon restored their courage. Dr. Reese heard the history of the transaction. He then threw water in the face of the sleeping negro, slapped him, raised him up, shook him violently, and after a little he was roused to consciousness, greatly to the relief of all present. The Doctor then gave the youngsters a lecture on the dangers of such frolics, and cautioned them against a repetition of their heedless act. This of course broke up the ether frolics in this neighborhood. Dr. Wilhite thinks it was more than an hour from the time the messenger started for Dr. Reese, till he returned with him to Mr. Ware's. The distance to Athens and back was ten miles, and all this time the negro boy was profoundly narcotized.

This is unquestionably the first case in which sulphuric ether was even given to the extent of producing complete anæsthesia.

Dr. Crawford W. Long, now of Athens, Georgia, was born in Danielsville, Madison County, Georgia, on the 1st November, 1815. He graduated at the University of Georgia (then the Franklin College) in 1835. He studied medicine and graduated at the Medical Department of the University of Pennsylvania in 1839. He then went to Jefferson, Jackson County, Georgia, where he practiced medicine for many years. In 1842, he had four students in his office, viz: P. A. Wilhite, John S. Groves, D. I. Long and H. R. P. Long. The two last were relatives of Dr. Long, and they are both dead. Wilhite and Groves are still living (1877). Dr. Long was 27 years old. His pupils were all from 19 to 21; they were on the best of terms with each other, the Doctor entering into all the sports of his pupils with a hearty good will, while he never neglected his duties as their teacher. On one occasion, they were talking about inhalation of nitrous oxide gas, when one of his pupils asked him to make some for them. He said he did not have suitable apparatus for it, but that the inhalation of sulphuric ether would produce precisely the same exhilarating effect. One of the young men present said he had inhaled ether while at school, and was willing to do it again. They were all anxious to witness its effects. Dr. Long got some ether immediately and gave it to the young man who had previously inhaled it. He then inhaled it himself, and afterwards gave it to all present. After this the young Doctor and his pupils indulged occasionally in ether frolics. On several occasions, Dr. Long became furiously excited and could not be controlled. On recovering from the ether intoxication, he frequently noticed that his arms and hands were badly bruised, and yet he was not conscious of having felt any pain at the time he was under the influence of the ether. He also noticed the same thing in his pupils. They were often badly hurt by falls and blows, and were not conscious of pain at the time. These facts, repeatedly observed, suggested to his mind the idea of using ether to prevent the pain of surgical operations. He frequently spoke of this to his students, and at last he determined to give it a trial. Willhite ecouraged him by relating the case of the negro boy he had playfully and unintentionally put under the influence of ether for an hour or more in the Fall of 1839.

Dr. Long having made up his mind to try the experiment with ether on the first favorable opportunity, says (*Southern Medical and Surgical Journal*, Dec., 1849 :)

"The first patient to whom I administered ether in a surgical operation, was Mr. James M. Venable, who then resided within two miles of Jefferson. Mr. Venable consulted me on several occasions with regard to the propriety of removing two small tumors situated on the back part of his neck, but would postpone from time to time having the operations performed, from dread of pain. At length I mentioned to him the fact of my receiving bruises while under the influence of the vapor of ether, without suffering, and, as I knew him to be fond of, and accustomed to inhale ether, I suggested to him the probability that the operations might be performed without pain, and proposed operating on him while under its influence. He consented to have one tumor removed, and the operation was performed the same day. The ether was given to Mr. Venable on a towel; and when fully under its influence I extirpated the tumor. It was encysted, and about half an inch in diameter. The patient continued to inhale ether during the time of the operation, and when informed it was over, seemed incredulous, till the tumor was shown him. He gave no evidence of suffering during the operation, and assured me, after it was over, that he did not experience the slightest degree of pain from its performance." *This operation was performed on the 30th of March*, 1842.

"The second operation I performed upon a patient etherized was on the 6th June, 1842, and was on the same person (Mr. Venable) for the removal of another small tumor. This operation required more time than the first, from the cyst of the tumor having formed adhesions to the surrounding parts. The patient was insensible to pain during the operation, until the last attachment of the cyst was separated, when he exhibited signs of slight suffering, but asserted after the operation was over that the sensation of pain was so slight as scarcely to be perceived. In this operation, the inhalation of ether ceased before the first incision was made."

In a certificate sworn to by James M. Venable on the 23d July, 1849, he says: "In the early part of the year (1842,) the young men of Jefferson and the country adjoining were in the habit of inhaling ether for its exhilarating powers, and I inhaled it myself frequently for that purpose and was very fond of its use. While attending the Academy, I was frequently in the office of Dr. C. W. Long, and having two tumors on the side and rather back of my neck, I several times spoke to him about the propriety of cutting them out, but postponed the operation from time to time. On one occasion, we had some conversation about the probability that the tumors might be cut out while I was under the influence of sulphuric ether, without my experiencing pain, and he proposed operating on me while under its influence. I agreed to have one tumor cut out, and had the operation performed that evening (afternoon) after school was dismissed. This was in the early part of the spring of 1842. I commenced inhaling the ether before the operation was commenced, and continued it until the operation was over. I did not feel the slightest pain from the operation, and could not believe the tumor was removed until it was shown to me. A month or two after this time, Dr. C. W. Long, cut out the other tumor situated on the same side of my neck. In this operation, I did not feel the least pain until the last cut was made, when I felt a little pain. In this operation, I stopped inhaling the ether before the operation was finished. I inhaled the ether in both instances from a towel, which was the common method of taking it."

Dr. Long's four students Wilhite, Groves, and the two Long's, also E. S. Rawls (now Dr. Rawls) and Andrew J. Thurmond, were present and assisted at the operation. Dr. Wilhite tells me that the etherization of Venable was as complete as it is ever made now-a-days, and that Venable always declared he felt no pain during the operation.

On the 3d July, 1842, Dr. Long amputated the toe of a negro boy, Jack, belonging to Mrs. Hemphill. Jack felt no pain, having been completely anæsthetized.

On the 9th September, 1843, Dr. Long exsected, without pain, three small cystic tumors from the head of Mrs. Mary Vincent, who was etherized for the purpose.

On the 8th January, 1845, Dr. Long amputated two fingers for a negro boy belonging to Mr. Ralph Bailey, Sr., the patient being fully etherized and feeling no pain whatever.

Morton's friends have been from the outset clamorous and persistent in proclaiming to the world "that Morton was the first man who ever produced complete anæsthesia for surgical operations." The facts above stated prove incontestably that they were mistaken; and before we get through it will be shown that they were doubly mistaken; for it will be established beyond controversy that Wells produced anæsthesia by nitrous oxide gas long before Morton did it with ether.

Long's anæsthesia with sulphuric ether was on the 30th March, 1842.

Well's anæsthesia with nitrous oxide gas was on the 11th December, 1844.

Morton's anæsthesia with sulphuric ether was on the 30th September, 1846.

Thus we see that Long ante-dates Wells two years and eight months, and ante-dates Morton four years and six months.

Dr. Long's operation under the influence of ether were known by all his neighbors—professional and non-professional. Many of these are still living. Dr. Wilhite lives at Anderson, South Carolina. Dr. John S. Groves, his fellow-student with Long in 1842, is now living at Dalton, Georgia. Dr. A. Delaperiere was the only physician, besides Dr. Long, at Jefferson in 1842, He witnessed these operations; has given his testimony to that effect, and is still living. Dr. E. S. Rawls, another witness, was living in Alabama a short time ago. All these men testify to the act that Long's operations under ether were witnessed and known by all medical men in his neighborhood and by the whole community.

Long's operations were not secret. He made no mystery about the substance given to prevent pain. He took out no patent for his discovery as did Morton and Jackson. He did not attempt to convert it into a money speculation. He published it before all men. It was not hidden from the world.

True, his was a very contracted world. He was waiting to test his great discovery in some capital operation. He lived in an obscure town where there were no railroads and no ponderous machinery to maim his fellow-men and the amputation of a leg or arm was an era in the life of a country doctor.

While he was still waiting for larger operations before communicating his discovery to some scientific journal, the labors of Wells and Morton and Jackson and Simpson burst upon the world. When Jackson made his visit to Long at Athens, in March, 1854, he said to Long: "You have the advantage of priority in date and in the first use of ether as an anæsthetic; but we have the advantage of priority of publication."

Now upon this point Long, Wells, Morton and Jackson stand individually upon the same level. Long exhibited to medical men and to the community his operations under ether (1842.) Wells exhibited to medical men and to the community his operation of the extraction of teeth under the influence of nitrous oxide gas (1844.) Morton exhibited to medical men and to the community the use of secret remedy, "Letheon," 1846 as anæsthetic. But Morton was fortunate in showing his patent remedy to the great surgeons of Boston. And it was not Morton, but it was Warren and Hayward and Bigelow who performed the operations at the Massachusetts General Hospital (October, 1846) on patients to whom Morton gave his "Letheon" that the world owes the immediate and universal use of anæsthesia in surgery. If Morton could have had his way he would have deodorized the ether and kept it a secret from the world.

Neither Wells nor Morton nor Jackson ever published a word on the subject till it burst forth in a blaze from the labors of the hospital surgeons already named.

When Warren and Hayward and Bigelow proved the real greatness of the discovery, then it was that Wells, Morton and Jackson began the war of pamphlets, and not till then did either of them publish in any scientific Journal a line about anæsthesia. And thus we see that its first publication to the world was really due to the illustrious surgeons of the Massachusetts General Hospital.

In 1853 Morton petitioned Congress to grant him a large sum of money for the discovery of anæsthesia. The friends of Wells opposed it, and claimed this honor for Wells, who used nitrous oxide gas as an anæsthetic two years and a half before Morton used ether for this purpose.

Then it was that the friends of Long appeared upon the scene, proving that Long was the first to used ether, antedating Morton four years and a half.

When Long's claim to the honor of discovering anæsthesia was presented to Congress by the Hon. Mr. Dawson, Senator from Georgia, it was formidable enough to block movements of Morton to get the appropriation he demanded for his discovery. They were so strong that Dr. Charles T. Jackson went to Athens, Georgia, expressly to see Dr. Long on the subject. In a communication to the Boston *Medical and Surgical Journal* April 11th, 1861, Dr. Charles T. Jackson says he visited Dr. Long at Athens, Georgia, on March 8th, 1854, to examine into Dr. Long's claims to being the first to use sulphuric ether as an anæsthetic in surgery, and he further says: "From the documents shown me by Dr. Long, it appears that he employed sulphuric ether as an anæsthetic agent—

*First.* On March 30th, 1842, when he extirpated a small glandular tumor from the neck of James M. Venable, a boy [Mr. Venable was over 21 years old when the operation was performed.—J. M. S.] in Jefferson, Georgia, now dead.

*Second.* On July 3d, 1842, in the amputation of the toe of a negro boy belonging to Mrs. Hemphill, of Jackson, Georgia.

*Third.* On September 9th, 1843, in the extirpation of a tumor from the head of Mary Vincent, of Jackson, Georgia.

*Fourth.* On January 8th, 1845, in the amputation of a finger of a negro boy belonging to Ralph Baily, of Jackson, Georgia.

Copies of the letters and depositions proving these operations with ether were all shown me by Dr. Long.

He also referred me to physicians in Jefferson who knew of the operations at the time.

The above extract from Dr. Jackson's paper from Boston *Medical Journal* recognizes Long's claim to being the first to produce anæsthesia for surgical operations, but it does not tell the whole story of Dr. Jackson's visit to Dr. Long.

Dr. Long has furnished me with all the evidence, consisting of affidavits, certificates, book entries, &c., that Dr. Jackson examined. He has also written me fully on the subject, and every fact that I have stated can be substantiated by documentary evidence.

In one of Dr. Long's letters to me (Nov. 5, 1876,) he says:

"In 1854 Dr Charles T. Jackson came to Georgia and spent two days with me in Athens, most of the time in my office, examining books, accounts, dates and certificates establishing the time, &c., of my operations. He expressed himself satisfied with the correctness of my claim to the first use of ether as an anæsthetic in surgical operations. Dr. Jackson informed me that he would go from Athens, to Dahlonega, Georgia, and as I knew he must pass through Jefferson where I resided up to 1850, and where my first operations under ether were performed, I requested him to stop in Jefferson and see some of the physicians there who witnessed or knew of the operations, and also a number of the citizens of the villege who either witnessed the operations or were familiar with them from common report. Dr. Jackson spent one or more days in Jefferson, and on his return, expressed himself satisfied with the testimony."

"In Dr. Jackson's communication to the Boston *Medical and Surgical Journal* (April 11th, 1861,) he neglected to say anything of the information he obtained while in Jefferson, although he admitted to me on his return, that the evidence was perfectly satisfactory."

The Hon. C. W. Andrews, of Madison, Georgia, informs me that he was in Dr. Long's employ and in his office when Dr. Jackson spent a whole day with Long in comparing notes and talking over the subject of etherization, and it seems that the real object of Dr. Jackson's visit to Dr. Long was to induce Long to unite with him in laying their conjoint claims before Congress as the real discoverers of anæsthesia as opposed to those of Morton. Jackson was willing to concede to Long the honor of being the first to use ether in surgical operations, but wished Long to concede to him the honor of priority in making the discovery of the principle of anæsthesia when he inhaled ether to relieve the pain and difficulty of breathing after inhaling chlorine gas (as Sir Humphrey Davy had done before.)

Dr. Long says (February 8th, 1877): "In our conversation I understood Dr. Jackson to yield the point of priority to me—and so did the Hon. C. W. Andrews. I did not admit to him that he was the first to make the discovery—leaving to me its practical application ; and when he proposed to me to unite our claims—he to claim the discovery and I its first practical use in surgical operations—I positively refused. I was satisfied that I was entitled to the credit of the discovery, as well as of the first practical use of ether in surgical operations."

"Instead of writing to Senator Dawson to unite our claims as Dr. Jackson requested, I wrote to Mr. Dawson to make no such compromise, but to place my claims solely on their merits; and if you will consult the Congressional proceedings of that time you will see that Mr. Dawson presented my claims separate and independent."

Now let us see how the followers of Long worked out the problem of anæsthesia without any knowledge whatever of his labors.

Horace Wells, a native of Hartford, Windsor County, Vermont, studied dentistry in Boston, and at the age of 21 (1836) he opened an office in Hartford, Connecticut, to practice his profession. His mind was early turned to the subject of preventing pain in the extraction of teeth. In August, 1840, Dr. L. P. Brockett, of Brooklyn, N. Y., then a medical student, went to Wells to have a molar tooth extracted ; the operation was difficult, and so painful that Wells said that there ought to be some method of mitigating such suffering, and that he thought a man might be made so drunk by the inhalation of nitrous oxide gas as to prevent the pain of dental and other operations. This shows how deeply impressed this subject was upon the mind of Wells at that early day. On December 10, 1844, Mr. G. Q. Colton delivered a lecture in Hartford, Conn., on "Laughing gas," and after the lecture he administered the gas to Wells and several other gentlemen. One of them (Mr. Cooley,) while under its influence, fell over some benches, and was evidently badly injured ; when he returned to consciousness, Wells rushed up to him and inquired if he was hurt. He replied, " No." Wells then said, " You must have been hurt, for you struck your legs against the benches " The young man then, at Well's suggestion, pulled up his pantaloons; the blood was running down his legs and his knees were badly injured. When again questioned by Wells, he said, " I did not feel any pain at the time." Wells then turned to a friend (Mr. David Clarke,) who was near by, and an eye witness to all of this, and remarked, " I believe a man by taking that gas could have a tooth extracted or a limb amputated and not feel the pain." So thoroughly was Wells convinced of this fact that he told his wife on their way home that he intended to take the gas the next day and have a tooth extracted. On arriving home, he left his wife and went to see his friend Dr. Riggs, to announce his great discovery, and his intention to take the gas for the extraction of a tooth. Riggs tried to dissuade him from it, but his mind was made up and he said, "As the young man did not feel pain at the time he was hurt, why cannot the gas be used in the extraction of teeth?" Early next morning (December 11) Wells called on Colton and engaged him to go to his office at ten o'clock and give him the gas. He did so, and Dr. Riggs extracted a large molar tooth for Wells while under the influence of the gas. Wells did not seem to feel any pain. He remained unconscious for a few moments, and on coming to, he exclaimed, "A new era in tooth-pulling! It did not hurt me more than the prick of a pin. It is the greatest discovery ever made."

From that moment Wells' enthusiasm was unbounded. He immediately began the administration of the gas, and daily extracted teeth under its influence; and other dentists in Hartford adopted the same practice with like success. Dr. Marcy, then of Hartford, on witnessing Wells' operations, told him that when a student at Amherst College, he, with other students had, for amusement, often inhaled nitrous oxide gas and also the vapor of sulphuric ether, and that the effects of the two were identical; and he suggested to Wells to try ether as a substitute for the gas. On this hint Wells tried it. He inhaled it himself, and he says, "I found it very difficult to inhale the vapor of ether, in consequence of the choking sensation. For this reason, and for the reason that Dr. Marcy and myself came to the conclusion that nitrous oxide gas was not so liable to do injury, I resolved to adhere to this alone."

About a month after the discovery of anæsthesia by Wells, Dr. Marcy (January, 1845) gave the vapor of sulphuric ether to a sailor for the extirpation of a small wen on the side of his head. The patient was insensible and the operation successful, but Marcy, after this experiment, still advised Wells to stick to the gas as being more agreeable, and perhaps, safer than ether. Wells continued the use of the gas, and the dentists (Riggs, Terry, Braddock and Crowfoot) and the doctors in Hartford were all convinced of its value as an anæsthetic. But Wells felt that his great discovery should be laid more broadly before the profession and the world, and early in 1845 went to Boston for this purpose. Through his former pupil and partner, Dr. Morton, dentist, he was introduced to Dr. John C. Warren, Dr. Charles T. Jackson, Dr. Hayward, and others. Dr. Warren received him kindly, and Wells remained in Boston several days with the expectation of giving the gas to a man who was to submit to an amputation at the hands of Dr. Warren. For some cause the operation was postponed. Wells was then invited to address the class at the medical college on the subject. He did so at some length, and then administered the gas for the extraction of a tooth. Unfortunately, the gas-bag was removed too soon; the patient was not sufficiently anæsthetized; he screamed out, and said he felt the pain of extraction, and the experiment was therefore a failure. Wells was hooted at, and unfeelingly hissed out of the amphitheatre by the thoughtless young men present, and he was pronounced a charlatan and his anæsthetic a humbug. He returned home greatly mortified at his failure, was taken suddenly ill and did not recover his health for many weeks.

In 1841-42, Morton was a pupil of Wells. In 1843, Wells established Morton in Boston, and for a while was his partner. In 1845-46, after Well's discovery of anæsthesia, by the use of nitrous oxide gas, they had frequent interviews, sometimes in Boston and sometimes in Hartford. After Wells unfortunate visit to Boston, Morton became greatly interested in the subject of anæsthesia. Notwithstanding Well's failures in Boston, Morton subsequently witnessed his continued success with the gas in Hartford, and was anxious to try it again in Boston. During one of his visits to Wells in Hartford in 1846, Morton asked Wells to show him how to make the gas. Wells not having time, referred him to Dr. Chas. T. Jackson to make it for him, as he was a chemist. On returning home, Morton called on Jackson for this purpose. Jackson told Morton that the manufacture of nitrous oxide gas required some nicety of manipulation; that there was danger of his getting nitric instead of nitrous oxide, and that he was too busy at that time to make it for him. Morton explained that he wished to use it to render patients insensible for the extraction of teeth. Jackson then told him to use the vapor of sulphuric ether, saying that it was perfectly safe, could be easily procured, and that the students at Cambridge often inhaled it for amusement.

[TO BE CONTINUED.]

